# Optimizing Nutrition to Counter Sarcopenia in Hepatocellular Carcinoma: A Narrative Review of Mechanisms, Clinical Consequences, and Supportive Therapeutic Options

**DOI:** 10.3390/nu18030494

**Published:** 2026-02-02

**Authors:** Hiroki Tai, Asahiro Morishita, Tomoko Tadokoro, Kyoko Oura, Rie Yano, Mai Nakahara, Koji Fujita, Shima Mimura, Joji Tani, Miwa Tatsuta, Takashi Himoto, Hideki Kobara

**Affiliations:** 1 Department of Gastroenterology and Neurology, Faculty of Medicine, Kagawa University, Kita-gun 761-0793, Kagawa, Japan; tai.hiroki@kagawa-u.ac.jp (H.T.); tadokoro.tomoko@kagawa-u.ac.jp (T.T.); oura.kyoko@kagawa-u.ac.jp (K.O.); yano.rie@kagawa-u.ac.jp (R.Y.); nakahara.mai@kagawa-u.ac.jp (M.N.); fujita.koji@kagawa-u.ac.jp (K.F.); mimura.shima@kagawa-u.ac.jp (S.M.); tani.joji.kb@kagawa-u.ac.jp (J.T.); kobara.hideki@kagawa-u.ac.jp (H.K.); 2 Department of Gastroenterology, KKR Takamatsu Hospital, 4-18 Tenjinmae, Takamatsu 760-0018, Kagawa, Japan; miwa114@gmail.com; 3 Department of Medical Technology, Kagawa Prefectural University of Health Sciences, Takamatsu 761-0123, Kagawa, Japan; himoto@kagawa-puhs.ac.jp

**Keywords:** hepatocellular carcinoma, sarcopenia, nutritional approach, exercise, quality of life

## Abstract

Patients with hepatocellular carcinoma (HCC) often sit at the crossroads of malignancy and chronic liver disease, where impaired hepatic reserve, systemic inflammation, and treatment-related stress accelerate loss of skeletal muscle mass and function. In this narrative review, we synthesize current evidence on the two-way relationship between sarcopenia and HCC management across curative and palliative settings. We outline key biological pathways—altered energy substrate use, amino acid imbalance, hyperammonemia-related signaling, and inflammatory and hormonal perturbations—that promote progressive muscle wasting, and we summarize how sarcopenia influences tolerance, complications, and outcomes of surgery, locoregional therapies, and systemic agents. We then translate the literature into practical supportive-care principles, including adequate energy and protein delivery, optimized meal distribution (including late-evening snacks), and selected supplementation alongside hepatic rehabilitation/exercise. Potential adjuncts discussed include branched-chain amino acids, L-carnitine, vitamin D, zinc, and other micronutrients. Because the available data are heterogeneous and largely derived from observational cohorts or extrapolated from cirrhosis populations, HCC-specific randomized trials and standardized intervention protocols remain limited. Therefore, nutritional and exercise recommendations should be individualized according to tumor stage, hepatic function, comorbidities, and treatment goals, and viewed as supportive guidance that requires confirmation in well-designed prospective studies.

## 1. Introduction

Hepatocellular carcinoma (HCC) is one of the leading causes of cancer-related mortality worldwide and typically arises in the setting of chronic liver disease and cirrhosis [1]. As a result, oncologic decision-making in HCC must contend not only with tumor burden but also with underlying hepatic dysfunction, portal hypertension, and systemic complications such as malnutrition and sarcopenia. Sarcopenia—defined as a progressive loss of skeletal muscle mass (SMM) and function—is now recognized as a prevalent and powerful prognostic factor in cirrhosis and HCC [2].

Several observational studies and meta-analyses have demonstrated that sarcopenia is associated with reduced overall survival, higher rates of treatment-related complications, and poorer tolerance to both locoregional and systemic therapies [3]. In patients undergoing potentially curative modalities such as surgical resection or ablation, sarcopenia predicts higher postoperative morbidity and mortality [4,5]. In those receiving transarterial chemoembolization (TACE) or systemic agents (e.g., multikinase inhibitors, immune checkpoint inhibitors [ICIs]), sarcopenia has been linked to therapy intolerance, early treatment discontinuation, and inferior outcomes [2].

In parallel, advances in the nutritional care of chronic liver disease have emphasized the central role of adequate energy and protein intake, timely meal distribution (including late-evening snacks), and targeted supplementation such as branched-chain amino acids (BCAA). Guidelines from the European Association for the Study of the Liver (EASL), the European Society for Clinical Nutrition and Metabolism (ESPEN), the American Association for the Study of Liver Diseases (AASLD), and other expert panels consistently highlight malnutrition, frailty, and sarcopenia as modifiable determinants of outcomes in cirrhosis [6,7,8].

Unlike reviews that primarily address cirrhosis in general or oncology cachexia broadly, the present review is designed to bridge three elements: (i) evidence that is explicitly linked to HCC stage and treatment modality (resection, transplantation, locoregional procedures, and systemic therapy); (ii) pragmatic nutrition and exercise strategies that can be implemented in day-to-day hepatology practice; and (iii) an appraisal of safety issues and key knowledge gaps to support individualized clinical decisions.

We also discuss several unresolved questions, including whether sarcopenia directly contributes to adverse outcomes or mainly reflects advanced hepatic dysfunction; how much muscle loss is driven by tumor-related cachexia versus cirrhosis-associated catabolism; the potential benefits and risks of high-protein diets and specific supplements in the context of hepatic metabolism and (theoretical) tumor biology; and whether clinically meaningful improvements typically require combined nutrition and structured exercise within hepatic rehabilitation programs rather than nutrition alone. Throughout, we separate well-established evidence from cirrhosis cohorts from the more limited, emerging data available in HCC populations.

Accordingly, this narrative review addresses the central question of how sarcopenia should be assessed and managed across the HCC treatment continuum, and which nutritional strategies are supported by evidence (and for whom), while explicitly acknowledging heterogeneity in definitions and the limited availability of HCC-specific randomized trials.

Patients with HCC represent a particularly vulnerable subgroup at the intersection of cancer cachexia, liver-related metabolic derangements, and treatment-induced catabolism. Nutritional therapy—including individualized dietary counseling, optimization of protein and energy intake, BCAA-enriched formulations, and micronutrient repletion—has therefore attracted growing interest as an adjunctive strategy to prevent or reverse sarcopenia, improve QOL, and potentially enhance survival in this population. In addition, structured exercise and “hepatic rehabilitation” programs are increasingly being integrated with nutritional support to synergistically counteract muscle wasting [9].

**Figure 1:**
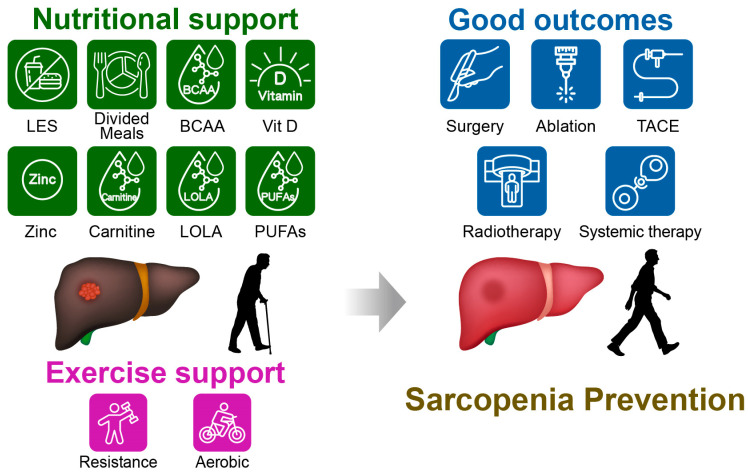
Conceptual summary of nutrition- and exercise-based support for sarcopenia in patients with hepatocellular carcinoma (HCC) and the clinical domains it may influence. Core dietary targets (adequate energy and protein intake, meal patterning including a late-evening snack, and focused supplementation) are illustrated alongside hepatic rehabilitation/exercise. Reported associations include improvements in physical performance, hepatic reserve, treatment tolerance, and quality of life. Examples of adjunctive options shown include branched-chain amino acids, vitamin D, zinc, L-carnitine, L-ornithine L-aspartate, and omega-3 polyunsaturated fatty acids. Representative HCC treatments are indicated, including surgery, ablation, transarterial chemoembolization, radiotherapy, and systemic therapy. Abbreviations: LES, late-evening snack; BCAA, branched-chain amino acids; Vit D, vitamin D; LOLA, L-ornithine L-aspartate; PUFAs, omega-3 polyunsaturated fatty acids; TACE, transarterial chemoembolization.

Figure 1 summarizes the nutritional and exercise support and associated clinical outcomes in sarcopenic patients with concomitant HCC. This narrative review summarizes current knowledge on the bidirectional interplay between HCC treatment and sarcopenia, with a particular focus on the role of nutritional interventions across curative and palliative settings. We first discuss the definition and assessment of sarcopenia in chronic liver disease and HCC and then outline the mechanistic links between hepatic dysfunction, systemic inflammation, and muscle depletion. We next review the impact of sarcopenia on outcomes of major HCC therapies. Finally, we show an in-depth examination of evidence supporting nutritional and exercise-based interventions.

## 2. Materials and Methods

We performed a focused search of PubMed/MEDLINE and Embase, complemented by hand-searching reference lists, to identify English-language studies and guidance documents addressing sarcopenia in chronic liver disease and HCC. The search window spanned January 2000 through November 2025 and combined terms such as “hepatocellular carcinoma”, “cirrhosis”, “sarcopenia”, “muscle mass”, “nutrition”, “branched-chain amino acids”, “late-evening snack”, “carnitine”, “vitamin D”, and “exercise/rehabilitation”. When available, we prioritized HCC-specific clinical evidence; otherwise, we incorporated higher-quality cirrhosis data and widely used consensus recommendations that are commonly applied to HCC care. Given the narrative format, we did not conduct a formal meta-analysis or structured risk-of-bias scoring. Instead, we explicitly note areas where evidence is indirect, inconsistent, or based on small observational cohorts.

## 3. Definition and Assessment of Sarcopenia in Chronic Liver Disease and HCC

### 3.1. General Definitions of Sarcopenia

The concept of sarcopenia has evolved from a focus on age-related muscle loss to a broader syndrome encompassing loss of muscle mass, strength, and physical performance, often driven by chronic disease. Major consensus groups such as the European Working Group on Sarcopenia in Older People and the Asian Working Group for Sarcopenia emphasize low muscle strength as the primary indicator, supported by measurements of muscle quantity/quality and functional performance [10,11]. In clinical practice, handgrip strength, chair stand tests, and gait speed are commonly used to assess muscle function, while dual-energy X-ray absorptiometry, bioelectrical impedance analysis (BIA), or cross-sectional imaging (computed tomography [CT]) are used to quantify muscle mass [12,13,14,15,16,17].

### 3.2. Liver Disease-Specific Definitions and Diagnostic Criteria

In liver diseases, the aging of the patient population has already been progressing, and advances in various therapeutic interventions have further prolonged survival, thereby accelerating this trend. Consequently, a substantial increase in the number of patients with concomitant sarcopenia is anticipated, and accumulating evidence indicates that the presence of sarcopenia is associated with poor clinical outcomes. Moreover, because the liver is the most essential metabolic organ, liver diseases readily induce secondary sarcopenia through mechanisms such as malnutrition [18]. Taken together, sarcopenia in patients with liver disease should be regarded as a distinct entity, referred to as “liver disease-related sarcopenia.” Guidelines from EASL, ESPEN, and AASLD recognize skeletal muscle loss as a key component of malnutrition and frailty in chronic liver disease [6,7,8]. Several hepatology societies and expert groups have proposed liver-specific diagnostic thresholds using CT-based skeletal muscle index (SMI) at the third lumbar vertebra (L3), psoas muscle index, or BIA-derived SMM [8,19]. These methods are particularly relevant in HCC, where CT imaging is routinely performed for staging and treatment planning.

While handgrip strength and gait speed are important prognostic indicators, their systematic assessment is not yet universally implemented in HCC clinical workflows. In some Japanese guidelines for liver disease nutrition, sarcopenia is diagnosed using a combination of SMM (assessed by CT or BIA) and handgrip strength, without mandating slow gait speed because low grip strength strongly predicts reduced walking speed [20]. This pragmatic approach facilitates integration into hepatology practice.

Many studies in HCC—especially those from Japan—adopt the Japan Society of Hepatology (JSH) definitions and region-specific cutoffs using CT-based indices or bioelectrical impedance analysis. Although these criteria are practical in routine care, differences among major consensus frameworks (e.g., EWGSOP2, AWGS, and JSH) complicate comparisons across populations. We therefore encourage authors to state clearly which definition and cutoffs were used and, when feasible, to include sensitivity analyses using alternative internationally recognized thresholds to enhance generalizability.

Because multiple diagnostic frameworks (e.g., EWGSOP2, AWGS, and liver disease-specific criteria such as the JSH consensus) coexist, direct comparison across studies can be challenging. In this review, we therefore emphasize transparent reporting of the applied definition (including cutoffs and measurement modality), and we encourage a pragmatic, tiered assessment in routine HCC practice: (i) screen for low muscle strength (e.g., handgrip), (ii) confirm low muscle quantity using an available modality (CT-based muscle area is often feasible in HCC given routine imaging), and (iii) assess physical performance when possible. Where feasible, sensitivity analyses using alternative region-appropriate cutoffs may improve generalizability and interpretability.

### 3.3. Prevalence of Sarcopenia in HCC

The reported worldwide prevalence of sarcopenia is 5–13% in persons aged in their 60s and 70s and 11–50% in persons over 80 years old [21,22,23]. In the USA, sarcopenia reportedly occurs in 40–70% of patients with cirrhosis, which is a higher prevalence than among patients with inflammatory bowel disease (approximately 20%) [23,24,25]. Sarcopenia is more frequent in advanced cirrhosis (Child–Pugh B/C), those with portosystemic shunts, and individuals with repeated decompensations (ascites, encephalopathy) [6]. Patients undergoing TACE or systemic therapy are at particularly high risk due to cumulative catabolic stress, reduced oral intake, and treatment-related adverse events [26,27].

### 3.4. Limitations and Challenges in Assessment

Despite increasing recognition, several challenges remain in the assessment of sarcopenia in HCC:

Heterogeneity of methods: Different cutoffs and imaging techniques limit comparability across studies [28].

Dynamic changes: Muscle mass and strength can change rapidly during cancer treatment, requiring serial assessment [27].

Confounding by fluid overload: Ascites and edema can obscure anthropometric measures and BIA [18].

Standardization of sarcopenia assessment in HCC, potentially using CT-based metrics available from routine imaging combined with simple functional measures, remains an important priority for both clinical practice and research [29].

## 4. Mechanistic Links Between Hepatic Dysfunction, Systemic Inflammation, and Muscle Loss

As shown in Table 1, liver dysfunction and sarcopenia are interconnected through multiple pathophysiological mechanisms.

### 4.1. Energy Metabolism and Substrate Utilization in Cirrhosis

Cirrhosis is characterized by profound alterations in energy metabolism, including decreased glycogen stores, early onset of fasting metabolism, and increased reliance on fat oxidation and gluconeogenesis from amino acids [30,31]. Patients often exhibit a “chronic fasting” state with increased resting energy expenditure, particularly in advanced disease. Inadequate caloric intake in this context accelerates proteolysis and muscle catabolism to meet energy demands [32]. Late-evening carbohydrate snacks and frequent meals have been shown to attenuate nocturnal protein breakdown and improve nitrogen balance [33,34].

### 4.2. Amino Acid Imbalance and the Role of BCAA

Valine, leucine, and isoleucine, which account for 40% of essential amino acids, are called BCAA. Skeletal muscle takes up BCAA, which are used for protein synthesis and also catabolized as an energy source [35,36]. Decreased BCAA levels are characteristic of decompensated cirrhosis and acute liver failure, which can lead to a reduction in muscle mass [36,37]. Furthermore, decreased BCAA concentrations in the blood and muscle may reduce ammonia clearance from the blood, potentially contributing to the progression of hepatic encephalopathy (HE) and sarcopenia [36,37,38].

Supplementation with BCAA has been reported to protect hepatic reserve capacity, suppress hepatocarcinogenesis in patients with decompensated cirrhosis and obesity, and reduce the cumulative recurrence rate after HCC treatment in patients with insulin resistance. Furthermore, it has also been reported to potentially contribute to improving sarcopenia in liver disease [39,40,41].

### 4.3. Hyperammonemia, Myostatin, and Skeletal Muscle Autophagy

Hyperammonemia is a hallmark of cirrhosis and has emerged as a key driver of muscle wasting [42]. Ammonia impairs mitochondrial function and inhibits protein synthesis [43]. In cirrhotic patients, higher ammonia levels have been associated with reduced muscle mass and strength as well as increased risk of HE and falls [44].

Myostatin is a protein that inhibits skeletal muscle growth and belongs to the transforming growth factor-β family [45]. Additionally, known by its gene name growth/differentiation factor-8 (GDF-8). In mice, deletion of the GDF-8 gene has been shown to increase muscle mass to 2–3 times the normal level [45]. In chronic diseases such as liver cirrhosis, myostatin concentrations in the blood and muscle tissue increase, contributing to a reduction in muscle mass [46]. Furthermore, hyperammonemia has been confirmed to increase myostatin transcription via nuclear factor-κB, thereby suppressing skeletal muscle protein synthesis [47].

### 4.4. Systemic Inflammation, Hormonal Changes, and Cancer-Related Factors

Systemic inflammation contributes to muscle wasting through elevated pro-inflammatory cytokines (e.g., tumor necrosis factor-alpha (TNF-α), interleukin-6 (IL-6)), activation of the ubiquitin–proteasome system, and resistance to anabolic stimuli [48,49,50]. HCC itself can exacerbate hypermetabolism and inflammation, further driving catabolism [51]. Hypogonadism, insulin resistance, and altered growth hormone (GH)/insulin-like growth factor-1 (IGF-1) signaling, commonly seen in cirrhosis, additionally impair muscle protein synthesis and promote adipose tissue redistribution [49,52,53,54,55,56].

Cancer therapies may also influence muscle mass. For example, tyrosine kinase inhibitors such as sorafenib and lenvatinib (LEN) are associated with appetite loss, diarrhea, and fatigue, which can reduce oral intake and physical activity [57]. Recent data suggest that LEN-related sarcopenia may be attenuated by levocarnitine supplementation in some patients [58].

**Table 1:** Mechanisms linking liver dysfunction to sarcopenia in patients with cirrhosis and HCC.

Mechanism	Key Pathophysiology	Clinical Impact	References
Energy metabolism abnormalities	Reduced glycogen stores Early onset of metabolism Gluconeogenesis from AA Chronic fasting state	↑ Proteolysis ↓ Muscle mass	[30,31,32,33,34]
Amino acid imbalance	Low BCAA High AAA	↑ HE risk ↓ Muscle protein synthesis	[36,37,38]
Hyperammonemia and myostatin	Mitochondrial dysfunction Myostatin upregulation	↑ Autophagy, proteolysis ↓ Muscle mass	[42,43,44,45,46,47]
Systemic inflammation	High TNF-α, IL-6 Ubiquitin–proteasome activation	↑ Catabolism, anorexia ↓ Physical function	[48,49,50]
Hormonal dysregulation	Low testosterone GH/IGF-1 axis impairment Insulin resistance	↑ Fat redistribution ↓ Muscle synthesis	[49,52,53,54,55,56]

AA, amino acids; BCAA, branched-chain amino acids; AAA, aromatic amino acids; HE, hepatic encephalopathy; TNF-α, tumor necrosis factor-alpha; IL-6, interleukin-6; GH, growth hormone; IGF-1, insulin-like growth factor-1. ↑ means increase or intensification, ↓ means decrease or weakening.

## 5. Impact of Sarcopenia on HCC Treatment Outcomes

When interpreting associations between sarcopenia and HCC outcomes, cohort differences and indication bias are important. Most reports are retrospective and vary by region, etiology (viral hepatitis versus metabolic dysfunction-associated steatotic liver disease/alcohol), baseline hepatic function, and treatment selection; more frail patients or those with advanced cirrhosis may be steered toward less intensive therapy, which can confound observed relationships. Where data permit, we emphasize studies using multivariable adjustment and propensity-based methods, while acknowledging that residual confounding is difficult to eliminate.

### 5.1. Surgical Resection and Liver Transplantation

Surgical resection is indicated for patients with solitary tumors or up to three tumors (tumor diameter ≤3 cm), no vascular invasion or extrahepatic metastasis, no portal hypertension, and good hepatic reserve, corresponding to Barcelona Clinic Liver Cancer (BCLC) stage 0 or A [59]. On the other hand, orthotopic liver transplantation generally requires meeting the Milan criteria (single tumor ≤5 cm in maximum diameter, or up to three tumors each ≤3 cm in multiple tumors, with no vascular invasion or extrahepatic metastasis). However, some institutions have expanded eligibility criteria to include alpha-fetoprotein levels and other oncological indicators [59,60].

In patients with resectable HCC, sarcopenia has been linked to higher rates of postoperative complications, infections, longer hospital stay, and reduced long-term survival. In a Japanese retrospective study, sarcopenia was demonstrated to predict increased short- and long-term postoperative complications and worse overall survival (OS) even independently of the preoperative risk as determined by the American Society of Anesthesiologists score [61]. Several retrospective series report that low preoperative SMI or psoas muscle area independently predicts postoperative liver failure and mortality, even after adjusting for tumor factors and hepatic reserve [5]. A study examining the association between OS and sarcopenia after liver resection in 174 Dutch HCC patients and 379 Japanese HCC patients found that sarcopenia was strongly associated with OS in the Japanese cohort, but no significant association was observed in the Dutch cohort. This indicates that risk assessment must account for regional differences [62]. Prehabilitation strategies combining nutritional optimization and exercise have been proposed to mitigate these risks, though high-quality prospective trials remain scarce [2].

In orthotopic liver transplantation, sarcopenia has been associated with increased wait-list mortality and poor post-transplant survival [19]. Malnourished or sarcopenic candidates may experience higher rates of infections, prolonged ventilation, and delayed graft function. Sarcopenia significantly increases the risk of short-term complications after liver resection and also contributes to prolonged hospital stays [63,64].

### 5.2. Locoregional Therapies: Ablation and TACE

Locoregional therapies include local ablation therapies such as radiofrequency ablation (RFA), microwave ablation (MWA), TACE, transarterial radioembolization (TARE), and stereotactic body radiotherapy (SBRT). Ablation is recommended for patients with BCLC stage 0 or A disease or when surgical resection is difficult [59]. TACE is recommended for patients with BCLC stage A, where ablation is difficult, and for patients with BCLC stage B [59]. TARE has reported efficacy for solitary tumors ≤8 cm with preserved hepatic reserve and performance status, while evidence for SBRT is limited and requires accumulation of prospective trials [59].

#### 5.2.1. Ablation

RFA utilizes the Joule heat generated by alternating current to thermally denature tumor tissue. MWA can induce coagulative necrosis through the heat generated by water molecules violently vibrating due to electromagnetic waves. Both methods carry the potential for complications such as fever, pain, and bleeding [65,66].

In a study of 56 patients with early-stage HCC who underwent RFA, 66.1% had sarcopenia. The median time to recurrence was 17.6 months in the sarcopenia group and 36.7 months in the non-sarcopenia group, showing a significant difference. Although sarcopenia was not identified as an independent risk factor for recurrence in multivariate analysis, it showed a trend toward increased recurrence risk [67].

#### 5.2.2. TACE

TACE involves injecting a chemotherapeutic agent, usually an anthracycline or cisplatin, along with embolic material such as lipiodol (conventional TACE) or non-resorbable embolic microspheres (drug-eluting beads TACE) into the vessels supplying the tumor mass, providing both a cytotoxic and an ischemic effect [68,69].

Increased risk of post-procedure complications: Sarcopenic patients may be more susceptible to infections, hepatic decompensation, and prolonged hospitalization after TACE [4].

Poor tolerance of repeated TACE: Repeated TACE sessions may exacerbate muscle wasting through cumulative reductions in appetite, fatigue, and subclinical inflammation [70].

Reduced survival: Multiple cohorts demonstrate that baseline sarcopenia is associated with worse overall and progression-free survival following TACE [27,71].

A prospective observational study of 152 HCC patients undergoing TACE showed that participation in a structured cancer rehabilitation program—combining supervised exercise and nutritional guidance—was associated with improved physical function and survival compared with standard care, suggesting that multimodal interventions may partially offset the negative impact of sarcopenia [9].

Even in transarterial embolization without the use of chemotherapeutic agents, sarcopenia negatively influences treatment outcomes, leading to higher mortality rates without an increase in adverse events or post-procedural complications [72].

#### 5.2.3. Radiotherapy

TARE is a local treatment for HCC and liver metastases that involves selectively delivering microspheres containing radioactive isotopes to tumors via the hepatic artery. The most commonly used isotope is yttrium-90, which delivers high-dose beta radiation to the tumor [73,74]. SBRT is a treatment that delivers high-dose radiation to the tumor in 1–5 fractions with high precision. It is gaining attention as a non-invasive option for unresectable HCC [75].

A decrease in PMI one month after TARE administration has been shown to be significantly associated with an increased incidence of subsequent progressive disease [76,77]. Furthermore, patients experiencing a decrease of 7% or more in SMI within 90 days after SBRT were shown to have significantly shorter OS compared to patients without such a decrease [76,78].

### 5.3. Systemic Therapies

Systemic therapy is indicated for patients with BCLC stage C disease or those who progressed after local therapy, particularly those with Child-Pugh A liver reserve [59]. Previously, the oral multi-kinase inhibitor sorafenib was considered standard treatment, with its antitumor effects based on anti-angiogenic activity and induction of apoptosis [79]. However, the IMbrave150 trial demonstrated that the combination therapy of atezolizumab and bevacizumab (ATZ/BEV) provided a significant survival benefit over sorafenib in untreated patients with unresectable HCC [80]. Currently, ATZ/BEV is recommended as first-line therapy for unresectable HCC, with sorafenib and lenvatinib positioned as alternative options [59].

Several studies have demonstrated that sarcopenia independently predicts poor outcomes in patients treated with sorafenib and other systemic therapies. In a recent multicenter study, sarcopenia at baseline was associated with reduced overall survival in patients receiving sorafenib, and its combination with a Model for End-Stage Liver Disease score >9 identified a subgroup with dismal prognosis, in particular [81]. Meta-analytic data further support that sarcopenia is associated with higher rates of dose reductions, treatment discontinuation, and early mortality in HCC patients receiving systemic agents [3].

In patients receiving combination therapy with ATZ/BEV, several studies have reported that the SMI measured by CT is a useful predictor of prognosis [82]. In addition, assessments of sarcopenia using SMI derived from BIA together with handgrip strength have also been shown to be prognostically informative [27]. Although sarcopenia is considered an important prognostic factor, only a limited number of studies have evaluated sarcopenia using both handgrip strength and SMI, and the sample sizes in these studies remain small.

Moreover, during systemic pharmacologic therapy, patients are prone to deterioration in nutritional status and declines in physical activity. Accordingly, it is desirable to perform nutritional assessments and monitor daily physical activity, and—when feasible—to consider incorporating exercise therapy in addition to nutritional interventions.

## 6. Principles of Nutritional Management in Cirrhosis and HCC

Table 2 provides a summary of the nutritional management strategies, which are described in detail below.

### 6.1. Energy Requirements

Patients with cirrhosis often exhibit increased resting energy expenditure, especially in decompensated disease. The ESPEN and EASL guidelines recommend an energy intake of approximately 30–35 kcal/kg/day for most patients with cirrhosis, adjusting for obesity, edema, and physical activity [6,7]. The recommended energy requirement for patients with impaired glucose tolerance is set at 25 kcal/kg/day [83]. In HCC patients undergoing active oncologic therapy, additional caloric needs may arise from treatment-related inflammation and reduced intake due to anorexia, nausea, or early satiety.

### 6.2. Protein Intake and Distribution

Contrary to historical dogma, protein restriction is no longer recommended in stable cirrhosis, even in those with prior HE. Current guidelines advocate a daily protein intake of 1.2–1.5 g/kg in patients with cirrhosis to prevent or reverse muscle loss [83]. Proteins should be distributed evenly across meals and snacks, with particular emphasis on a late-evening carbohydrate- and protein-containing snack to counteract nocturnal catabolism. Plant-based proteins and BCAA-enriched formulations may be especially beneficial in patients with encephalopathy or intolerance to animal protein [41].

### 6.3. Meal Timing and Late-Evening Snacks

A late-evening snack (LES) is a nutritional intervention in which approximately 200 kcal of the recommended daily energy intake is consumed before bedtime. In patients with LC, enhanced catabolism makes them prone to malnutrition even after short periods of fasting. Therefore, to ameliorate fasting-induced metabolic deterioration, divided meals consisting of 4 to 7 feedings per day, including an LES, are recommended [84]. Case–control studies have also reported that LES contributes to improved clinical outcomes in patients with LC [85]. Individualized dietary counseling should incorporate education on meal frequency, timing, and composition to sustain adequate energy and protein delivery across the 24 h cycle [86].

## 7. BCAA Supplementation in Cirrhosis and HCC

Data supporting BCAA supplementation specifically in HCC remains limited and mixed. Most evidence comes from observational studies or small interventional cohorts, so reported advantages—such as better albumin maintenance, attenuated muscle loss, or improved treatment continuity—should be interpreted cautiously until larger, HCC-focused randomized trials are available.

### 7.1. Pharmacological Effects

BCAA are not only effective in improving nutritional status but also function as pharmacological nutrients with diverse biological actions [87,88]. They promote liver regeneration by acting on hepatic stellate cells and enhancing the secretion of hepatocyte growth factor, while simultaneously suppressing the expression of transforming growth factor-β receptors in these cells, thereby exerting inhibitory effects on the progression of hepatic fibrosis [89,90].

### 7.2. Evidence in Cirrhosis

In advanced cirrhosis, long-term oral BCAA granules (typically 0.25 g/kg/day) have been shown to improve event-free survival, reduce hepatic decompensation, and increase serum albumin concentrations compared with isoenergetic controls [91]. A large prospective study also identified amino acid imbalance (low BCAA-to-tyrosine ratio) as a risk factor for HCC development and demonstrated that BCAA supplementation reduced the incidence of HCC and prolonged survival in cirrhotic patients, particularly in those with obesity or higher body weight [41].

Recent systematic reviews and meta-analyses confirm that BCAA supplementation in cirrhosis improves albumin levels, reduces ascites and encephalopathy, and may decrease mortality, especially when administered for at least 6 months [92].

### 7.3. Evidence in HCC and Post-Treatment Settings

BCAA have been reported to improve serum albumin levels, QOL, HCC recurrence, and overall prognosis in patients treated with local therapies [92,93,94,95]. In addition, BCAA have been shown to enhance hepatic functional reserve and prognosis in HCC patients treated with molecular targeted agents (MTAs), as well as to alleviate LEN-associated fatigue [96,97,98]. BCAA have also been reported to increase the sensitivity of HCC stem cells to 5-fluorouracil [99]. Furthermore, BCAA activate natural killer cells through the induction of interleukin-12 secretion from dendritic cells and enhance the function of CD8-positive T cells, thereby augmenting the therapeutic efficacy of anti-PD-1 antibodies [100,101]. These findings suggest that BCAA may exert synergistic effects during immunotherapy.

### 7.4. Practical Considerations

Dose and formulation: Typical oral preparations deliver 12–14 g BCAA/day in divided doses; palatability and gastrointestinal tolerance should be considered.

Timing: BCAA may be taken between meals and/or as part of the late-evening snack to maximize anabolic signaling and minimize overnight catabolism.

Monitoring: Periodic assessment of albumin, BCAA-to-tyrosine ratio (where available), and patient-reported tolerance is recommended.

BCAA supplementation should be seen as a complement rather than a substitute for achieving the target total protein intake of 1.2–1.5 g/kg/day [7].

## 8. Other Nutritional and Pharmacologic Strategies to Counteract Sarcopenia

Before recommending adjunctive agents (e.g., L-carnitine, vitamin D, zinc, L-ornithine L-aspartate, or omega-3 polyunsaturated fatty acids), clinicians should consider feasibility factors such as adherence, pill burden, and cost, and should monitor for adverse effects (e.g., gastrointestinal symptoms, hypercalcemia with excessive vitamin D dosing, and potential interactions with concurrent medications). Whenever possible, supportive interventions should be tailored to tumor stage, treatment strategy, and hepatic reserve, and communicated as symptom- and function-oriented care rather than definitive oncologic therapy.

### 8.1. Vitamin D (Vit D)

Vit D is best known for its role in calcium balance and skeletal health; deficiency can cause rickets in children and osteomalacia in adults. However, Vit D also has broader biological actions, including effects on cell proliferation and differentiation [102]. It contributes to lymphocyte activation and immune regulation, and observational and experimental studies suggest anti-inflammatory and anti-fibrotic properties. These pleiotropic effects have led to interest in Vit D across a range of conditions, including infections, autoimmune and cardiovascular diseases, degenerative disorders, and cancers [103].

The liver is a key site for Vit D metabolism, including conversion to 25-hydroxy Vit D [104]; accordingly, low Vit D levels are common in chronic liver disease. Deficiency has been associated with reduced muscle strength and increased fall risk [105]. Some studies suggest that supplementation may be linked to improved outcomes in decompensated liver cirrhosis, whereas evidence for benefits on HCC-related endpoints or muscle strength remains limited [103]. In practice, when deficiency is identified, Vit D repletion is reasonable as part of a broader strategy that also ensures adequate protein intake and incorporates exercise as tolerated.

From a clinical standpoint, Vit D should generally be viewed as a correction of a common deficiency rather than a proven anabolic therapy. Recent evidence syntheses in chronic liver disease suggest that supplementation may modestly improve selected biochemical or metabolic parameters, but clinically meaningful endpoints and muscle-related outcomes remain uncertain and heterogeneous across trials [106]. In cirrhosis-focused comparative analyses, combined nutritional strategies such as BCAA plus Vit D have shown signals of benefit for muscle indices, yet the overall certainty of evidence is low and not specific to HCC [107]. Therefore, we recommend measuring 25-hydroxyvitamin D when deficiency is suspected, supplementing to achieve guideline-recommended target levels, and monitoring for adverse effects (e.g., hypercalcemia or nephrolithiasis), particularly in patients receiving calcium or with renal dysfunction.

### 8.2. Zinc

Zinc is an essential trace element required for normal cellular growth, development, and differentiation. It is involved in DNA synthesis, RNA transcription, cell division, and cellular activation [108]. Zinc also constitutes a critical component of numerous zinc-binding proteins and enzymes, including key zinc-dependent transcription factors such as hepatocyte nuclear factor-4α, which is indispensable for hepatocellular function [109]. In addition, zinc plays an important role in ammonium metabolism and ammonia detoxification in both skeletal muscle and the liver [110].

Zinc deficiency or dysregulated zinc metabolism is frequently observed in various liver diseases. The mechanisms underlying zinc deficiency include reduced dietary intake, increased urinary excretion, activation of specific zinc transporters, and induction of hepatic metallothionein [108,111]. Zinc deficiency is particularly common in patients with LC, with some studies reporting a prevalence as high as 84–96% [108,112]. Moreover, zinc deficiency has been shown to correlate with the severity of liver disease, susceptibility to infections, reduced post-transplant survival, and an increased risk of malnutrition [113,114]. Zinc supplementation has been reported to inhibit or attenuate alcoholic liver injury through multiple mechanisms, including stabilization of the intestinal barrier function, reduction in endotoxemia, suppression of pro-inflammatory cytokine production, mitigation of oxidative stress, and inhibition of hepatocyte apoptosis [115].

Clinical manifestations of zinc deficiency include dermatologic lesions, dysgeusia, and muscle cramps. Severe zinc deficiency itself has been reported to cause neuropsychiatric disturbances and HE. Because zinc metabolism is closely linked to ammonia metabolism, zinc supplementation has been shown to improve hepatic nitrogen clearance in patients with advanced cirrhosis [116,117].

Taken together, zinc supplementation may improve appetite, cognitive function, and ammonia metabolism, thereby indirectly supporting adequate nutritional intake. Hypozincemia is a known risk factor for hepatocarcinogenesis, and zinc acetate hydrate, which improves this pathological condition, has been reported to exert inhibitory effects on the development of liver cancer [118,119,120]. When zinc supplementation is administered, approximately 50 mg/day of elemental zinc should be taken orally with meals to minimize potential adverse effects such as nausea and copper deficiency [115].

### 8.3. Carnitine

L-carnitine (4-N-trimethyl ammonium 3-hydroxybutyric acid) is a conditionally essential amino acid that is synthesized from the essential amino acids methionine and lysine in the human liver, kidneys, and brain, although it is primarily obtained through dietary intake [121]. More than 95% of total body carnitine is believed to be stored in skeletal muscle [122,123]. L-carnitine facilitates the transport of long-chain fatty acids across the mitochondrial membrane, thereby enabling their oxidative degradation for energy production [124]. Deficiency of L-carnitine is associated with LC due to reduced dietary intake, impaired absorption, and diminished endogenous hepatic synthesis [125].

Supplementation with carnitine may serve as an important therapeutic strategy to improve the quality of life in cirrhotic patients, particularly those experiencing sarcopenia, muscle cramps, or HE [126]. Supplementation with levocarnitine has been reported to attenuate the decline in hepatic functional reserve associated with TACE and to improve LEN-related sarcopenia [127,128].

### 8.4. L-Ornithine L-Aspartate (LOLA)

LOLA is routinely used in clinical practice to effectively reduce circulating ammonia levels in patients with LC [129]. Previous studies have demonstrated that LOLA lowers blood ammonia concentrations in rats with hyperammonemia induced by end-to-side portacaval shunting (PCA). Furthermore, administration of LOLA to PCA rats, in combination with rifaximin, markedly reduced ammonia levels in both the circulation and skeletal muscle, leading to significant improvements in lean body mass, grip strength, SMM, and muscle fiber diameter [130]. The rate of protein synthesis in the gastrocnemius muscle, which is significantly reduced after PCA, was also restored by ammonia-lowering interventions.

Across studies in experimental models of HE and in patients with cirrhosis and hyperammonemia, LOLA has consistently been shown to reduce circulating ammonia and ameliorate the severity of HE [131,132]. These effects involve mechanisms operating in both the liver and skeletal muscle. Because L-ornithine is a key intermediate of the urea cycle, it enhances the conversion of ammonia to urea by residual periportal hepatocytes [131]. Simultaneously, transamination of L-ornithine yields glutamate, the essential substrate for glutamine synthetase, thereby promoting glutamine synthetase-mediated conversion of ammonia to glutamine primarily in skeletal muscle [131,133]. Through these two independent pathways—hepatic urea synthesis and muscular glutamine synthesis—LOLA reduces ammonia levels in both blood and muscle, improves skeletal muscle phenotype and function, and mitigates ammonia-induced molecular disturbances [130].

In addition to its ammonia-lowering properties, other mechanisms may contribute to the beneficial effects of LOLA on sarcopenia in cirrhosis. An increasing body of evidence suggests that LOLA exerts hepatoprotective effects in patients with LC. This evidence, derived from multiple clinical trials, includes improvements in circulating liver transaminases, bilirubin, and prothrombin time [134]. Improvements in Child–Pugh and Model for End-Stage Liver Disease scores have also been reported in patients with chronic liver disease treated with LOLA, accompanied by significant reductions in circulating ammonia and enhanced cognitive function [135]. Proposed mechanisms underlying the hepatoprotective actions of LOLA include antioxidant effects mediated by increased glutathione synthesis—derived from the transamination of L-ornithine to glutamate—and improved hepatic microcirculation resulting from enhanced nitric oxide (NO) synthesis, attributable to increased production of L-arginine, an essential substrate for NO synthase [136,137]. Although further research is needed to clarify whether NO contributes to the pathogenesis of sarcopenia in cirrhosis, increased NO production in muscle has been reported to induce S-nitrosylation of calpain and attenuate age-related sarcopenia progression [138].

LOLA has the capacity to restore feeding-induced muscle protein synthesis in patients with cirrhosis. The mechanisms by which LOLA benefits sarcopenia in this population are primarily linked to its ammonia-lowering effects. Additionally, LOLA exerts hepatoprotective actions by providing key substrates—including glutamine, the antioxidant glutathione, and L-arginine, the substrate for nitric oxide synthase—through metabolic conversion of L-ornithine.

### 8.5. Omega-3 Polyunsaturated Fatty Acids (PUFAs) and Anti-Inflammatory Strategies

PUFAs, including eicosapentaenoic acid (EPA) and docosahexaenoic acid (DHA), play essential roles in human health, one of their primary functions being the attenuation and resolution of inflammation [139]. Supplementation with PUFAs has been reported to improve SMM and to be beneficial in preventing sarcopenia [140]. Randomized clinical trials have demonstrated that fish oil-derived PUFAs therapy increases muscle mass and strength in healthy older adults compared with control groups [141]. Among patients with cancer, circulating omega-3 fatty acid levels at baseline and during chemotherapy for lung cancer have been shown to correlate with sarcopenia and muscle loss [142]. Furthermore, in patients with gastrointestinal cancers undergoing systemic chemotherapy, nutritional supplementation enriched with fish oil has been reported to improve muscle mass and lean body mass [143].

In patients with HCC, both before and after propensity score matching, preoperative sarcopenia was associated with lower levels of EPA and DHA. These findings suggest that PUFAs may represent a promising nutritional intervention for HCC patients with sarcopenia. Further research and clinical trials investigating PUFAs supplementation in this patient population are warranted [144].

**Table 2:** Nutritional interventions for sarcopenia in patients with HCC.

Intervention	Recommendation	Note	References
Energy intake	30–35 kcal/kg/day 25 kcal/kg/day (DM)	Prevent catabolism	[6,7,83]
Protein intake	1.2–1.5 g/kg/day	Prevent muscle loss	[83]
Divided meals	From 4 to 7 times/day	Improvement of starvation status	[84]
LES	200 kcal/day before bedtime	Improvement of starvation status	[84,85]
BCAA	12–14 g/day intake	Improvement of AA imbalance Prolonged survival of HCC patients	[41,91,92,93,94,95,96,97,98,99,100,101]
Vit D	Supplementation of cholecalciferol	Linked to muscle weakness and falls Limited HCC evidence	[103,105,106,107]
Zinc	50 mg/day of zinc acetate hydrate	Improvement of appetite and ammonia metabolism by hypozincemia Prevent the occurrence of HCC	[115,116,117,118,119,120]
Carnitine	Supplementation of levocarnitine	Improvement of mitochondrial function Enhancement of QOL among patients with LC Mitigation of TACE-related deterioration in hepatic function Improvement of LEN-associated sarcopenia	[124,125,126,127,128]
LOLA	Oral LOLA	Antioxidant effects and improved hepatic microcirculation Hepatoprotective effects in LC	[134,135,136,137,138]
PUFAs	Supplementation of PUFAs	Anti-inflammation Associated with lower levels of PUFAs in sarcopenia with HCC	[139,140,141,142,143,144]

LES, late-evening snack; BCAA, branched-chain amino acids; AA, amino acids; HCC, hepatocellular carcinoma; Vit D, vitamin D; QOL, quality of life; LC, liver cirrhosis; TACE, transarterial chemoembolization; LEN, lenvatinib; LOLA, L ornithine L aspartate; PUFAs, omega-3 polyunsaturated fatty acids.

## 9. Exercise Management in Cirrhosis and HCC

Exercise prescriptions in cirrhosis and HCC should be guided by safety and feasibility. Particular caution is warranted in patients with decompensated disease, marked portal hypertension, refractory ascites, high variceal bleeding risk, or severe frailty. In such circumstances, programs should be individualized (often lower-intensity and supervised) and paired with nutritional optimization. Evidence for structured hepatic rehabilitation specifically in HCC is still developing.

In 2025, a feasibility study described a structured, multidimensional exercise program for cirrhosis-associated sarcopenia and reported improvements in muscle mass, strength, physical function, and quality of life without major safety signals [145]. These findings support the practicality of program-based “hepatic rehabilitation” approaches that could be adapted and evaluated in HCC cohorts.

### 9.1. About “Hepatic Rehabilitation”

Sarcopenia is an important clinical condition that affects both prognosis and QOL in patients with HCC. In April 2023, the Japan Society of Hepatology published the Guidelines for “hepatic rehabilitation”, recommending the implementation of rehabilitation programs for patients with HCC [146]. Although the mechanisms by which rehabilitation improves clinical outcomes in HCC patients have not yet been fully elucidated, exercise has been reported to ameliorate intratumoral hypoxia through modulation of hypoxia-inducible factor-1α, and to suppress hepatic cancer stem cells via the Akt/glycogen synthase kinase-3β/β-catenin signaling pathway [147]. Furthermore, myokines secreted from muscle cells during contraction have been shown to activate immune cells and exert antitumor effects, and exercise has been demonstrated to increase circulating chemokines with antitumor activity [148,149,150].

Recent meta-analyses have reported that combined aerobic and resistance exercise reduces the incidence of severe events, including HCC, in patients with LC [151]. Given that patients with chronic liver disease are at high risk for sarcopenia, exercise regimens incorporating resistance training—which has strong preventive and therapeutic effects on sarcopenia—are considered desirable. However, physical function and hepatic reserve vary widely among individuals, and adverse events such as falls, cardiovascular complications, renal dysfunction, and worsening HE have been reported in association with exercise therapy [151]. Therefore, it is essential to design individualized exercise programs and to implement rehabilitation through multidisciplinary collaboration [9].

### 9.2. Exercise Effects on Sarcopenia in HCC Treatment

Although evidence regarding the efficacy of exercise therapy in patients with HCC remains limited, a randomized controlled trial involving patients scheduled for hepatic surgery reported that exercise therapy improved insulin resistance associated with hepatic dysfunction and facilitated earlier postoperative resumption of physical activity [152]. Furthermore, exercise therapy has been shown to attenuate muscle atrophy in HCC patients hospitalized for TACE or treatment with MTAs, as well as to improve painful muscle cramps and frailty [153,154,155,156].

## 10. Future Directions and Research Priorities

Newer studies published in 2025 highlight the need for more rigorous interventional research. For instance, a randomized trial in major liver surgery found that a 6-week multimodal prehabilitation strategy—combining structured exercise with nutritional supplementation—reduced postoperative morbidity and improved muscle-related indices in sarcopenic patients [157]. Although not limited to HCC, these results support the feasibility and potential clinical value of integrated interventions in hepatobiliary oncology pathways. Concurrently, recent imaging-based work has improved prognostic stratification by combining measures of muscle quantity/quality with malnutrition metrics in primary HCC and by characterizing body composition in patients receiving combined locoregional and systemic therapy [158,159]. Future HCC-specific trials should standardize sarcopenia definitions, include patient-reported outcomes, and test scalable “nutrition plus exercise” models across etiologies and regions.

In the perioperative setting, a notable example is the PREHEP randomized clinical trial, in which a 6-week multimodal program combining structured exercise with nutritional supplementation (including BCAA) reduced 90-day postoperative morbidity in sarcopenic patients undergoing major hepatectomy [157]. Moreover, recent systematic reviews and meta-analyses in cirrhosis support the concept that combined exercise and nutrition interventions can improve muscle-related parameters, although intervention content, duration, and outcomes vary substantially [160,161].

Despite increasing attention to the relationship between HCC, sarcopenia, and nutritional therapy, several important challenges remain unresolved.

(i) Standard diagnostic criteria for sarcopenia in patients with HCC:

Although diagnostic criteria for sarcopenia in chronic liver disease have been proposed, HCC-specific criteria have not yet been clearly established. Only a limited number of studies have evaluated sarcopenia using both handgrip strength and SMI, and most of these reports involve small sample sizes. Further investigations are therefore required to define standardized diagnostic approaches tailored to HCC populations.

(ii) Randomized trials of nutritional interventions in HCC:

Most studies on BCAA and nutritional therapy have focused on patients with LC rather than HCC-specific cohorts. There is a need for randomized controlled trials that evaluate stratified nutritional interventions according to hepatic functional reserve and specific HCC treatment modalities.

(iii) Interactions between nutritional therapy, the tumor immune microenvironment, and systemic therapies:

Nutritional interventions may influence the tumor immune microenvironment and thereby modulate the efficacy and toxicity of ICIs and MTAs. Elucidating these underlying mechanisms will require further mechanistic and clinical research.

(iv) Artificial intelligence (AI) and sarcopenia assessment:

AI-based systems capable of automatically quantifying muscle and fat mass from CT imaging, as well as AI-assisted ultrasound analysis for standardized muscle quality assessment, have already been reported [162,163]. Applying these technologies to HCC populations to evaluate sarcopenia and to determine its impact on various treatment outcomes represents a promising future research direction.

## 11. Conclusions

Sarcopenia is highly prevalent in patients with HCC and exerts a profound influence on treatment tolerance, complications, and survival across the spectrum of curative and palliative therapies. Driven by a complex interplay of hepatic dysfunction, hyperammonemia, systemic inflammation, hormonal alterations, and treatment-related factors, muscle depletion represents a modifiable target for intervention.

Nutritional management—centered on meeting energy and protein targets, optimizing meal timing, and using selected supplements when appropriate—has a solid evidence base in cirrhosis, but only limited and heterogeneous support in HCC populations. Therefore, in HCC, the anticipated benefits should be described primarily as supportive (e.g., preserving function, hepatic reserve, and treatment delivery) rather than as proven improvements in oncologic endpoints. Well-designed, HCC-focused randomized trials with standardized definitions and clinically meaningful outcomes are needed to clarify the effectiveness, safety, and cost-effectiveness of individual strategies.

Multidisciplinary care pathways that incorporate routine screening for sarcopenia, individualized dietary counseling, and appropriately tailored hepatic rehabilitation/exercise—while accounting for contraindications in decompensated disease—may improve quality of life and help maintain treatment intensity. Key research priorities include identifying which subgroups (by etiology, stage, and treatment type) benefit most and determining how to deliver scalable interventions across diverse healthcare settings.

## Data Availability

No new data were created or analyzed in this study. Data sharing is not applicable to this article.

## Abbreviations

AA	amino acids
AAA	aromatic amino acids
AASLD	American Association for the Study of Liver Diseases
AI	Artificial intelligence
ATZ/BEV	atezolizumab/bevacizumab
BCAA	branched-chain amino acids
BCLC	Barcelona Clinic Liver Cancer
BIA	bioelectrical impedance analysis
CT	computed tomography
DHA	docosahexaenoic acid
DM	diabetes mellitus
EASL	European Association for the Study of the Liver
EPA	eicosapentaenoic acid
ESPEN	European Society for Clinical Nutrition and Metabolism
GDF-8	growth/differentiation factor-8
GH	growth hormone
HCC	hepatocellular carcinoma
HE	hepatic encephalopathy
ICIs	immune checkpoint inhibitors
IGF-1	insulin-like growth factor-1
IL-6	interleukin-6
LC	liver cirrhosis
LEN	lenvatinib
LES	late-evening snack
LOLA	L-ornithine L-aspartate
MTAs	molecular targeted agents
MWA	microwave ablation
OS	overall survival
PCA	portacaval shunting
PUFAs	omega-3 polyunsaturated fatty acids
QOL	quality of life
RFA	radiofrequency ablation
SBRT	stereotactic body radiotherapy
SMI	skeletal muscle index
SMM	skeletal muscle mass
TACE	transarterial chemoembolization
TARE	transarterial radioembolization
TKIs	tyrosine kinase inhibitors
TNF-α	tumor necrosis factor-alpha
Vit D	vitamin D
